# Optimization, purification and characterization of laccase from *Ganoderma leucocontextum* along with its phylogenetic relationship

**DOI:** 10.1038/s41598-022-06111-z

**Published:** 2022-02-14

**Authors:** Aisha Umar, Shakil Ahmed

**Affiliations:** Institute of Botany, University of the Punjab, Lahore, Pakistan

**Keywords:** Biochemistry, Biotechnology, Microbiology

## Abstract

The aim of this work to study an efficient laccase producing fungus *Ganoderma leucocontextum,* which was identified by ITS regions of DNA and phylogenetic tree was constructed. This study showed the laccase first-time from *G. leucocontextum* by using medium containing guaiacol. The growth cultural (pH, temperature, incubation days, rpm) and nutritional (carbon and nitrogen sources) conditions were optimized, which enhanced the enzyme production up to 4.5-folds. Laccase production increased 855 U/L at 40 °C. The pH 5.0 was suitable for laccase secretion (2517 U/L) on the 7th day of incubation at 100 rpm (698.3 U/L). Glucose and sucrose were good carbon source to enhance the laccase synthesis. The 10 g/L beef (4671 U/L) and yeast extract (5776 U/L) were the best nitrogen source for laccase secretion from *G. leucocontextum.* The laccase was purified from the 80% ammonium sulphate precipitations of protein identified by nucleotides sequence. The molecular weight (65.0 kDa) of purified laccase was identified through SDS and native PAGE entitled as Glacc110. The Glacc110 was characterized under different parameters. It retained > 90% of its activity for 16 min incubation at 60 °C in acidic medium (pH 4.0). This enzyme exerted its optimal activity at pH 3.0 and temperature 70 °C with guaiacol substrate. The catalytic parameters *K*_*m*_ and *V*_*max*_ was 1.658 (mM) and 2.452 **(**mM/min), respectively. The thermo stability of the laccase produced by submerged fermentation of *G. leucocontextum* has potential for industrial and biotechnology applications. The results remarked the *G. leucocontextum* is a good source for laccase production.

## Introduction

*Ganoderma* P. Karst. (Ganodermataceae) belongs to order Polyporales^1^. The species *Ganoderma leucocontextum* T.H. Li, W.Q. Deng, Sheng H. Wu, Dong M. Wang & H.P. Hu (Ganodermataceae) is commonly called ‘Zanglingzhi” or “White Lingzhi” in China^2^. This health oriented herbal mushroom contains numerous pharmacological bioactive secondary compounds, which are important in pharmacology due to therapeutic effects especially in America, Europe and China^3–6^. The reason of limited investigation on *G. leucocontextum* is scarceness of this species in the world^2^.

The world attention is moved around its ligninolytic laccase production and its applications^7^. Laccase, an extracellular isozyme belongs to family oxidoreductase^8^. This is ecofriendly green catalyst released molecular oxygen during the mechanism and flexible to accommodate the different substrates^9,10^, *e.g.,* diphenols, polyaromatic amines, iodine phosphates, ketones, ascorbate and lignin^11,12^. This biocatalyst is valuable at industrial level and also useful in textiles and biofuel production^13^. The major applications in the field of medicine are anticancerous drugs, hormone derivatives, preparation of antiviral agents, and antioxidants preparations^14^. The oxidative coupling reactions are also catalyzed by fungal laccase.

This enzyme plays a vital role in the delignification of plant products, fungal sporulation, conidial pigmentation and fruiting body formation of mushrooms^15,16^. This mushroom secreted metalloenzymes (laccase) possess “Janus-faced” range of activities *e.g.,* humification, azodye oxidation, xenobiotic compounds degradation^17,18^, phenolics, non-phenolics, pollutants detoxification^19^, wine and water discoloration, paper processing, steroid transformation, polymerization or depolymerization processes, biochemical bleaching of pulp, pharmaceutical products synthesis and degradation^20^, producer of other enzyme, biosensors constructor, and also play an important role in nanotechnology^21^.

In this study, first time *G. leucocontextum* is collected from Pakistan and identified by ITS marker^22^. The laccase is optimized to maximize the laccase potential by economical submerge fermentation techniques. This indicated that *G. leucocontextum* is a good source for laccase synthesis. We also reported here the biochemical characterization of laccase for industrial applications, where highly theromostable enzymes are required for multipurposes.

## Materials and methods

### Collection of specimen

**Figure 1:**
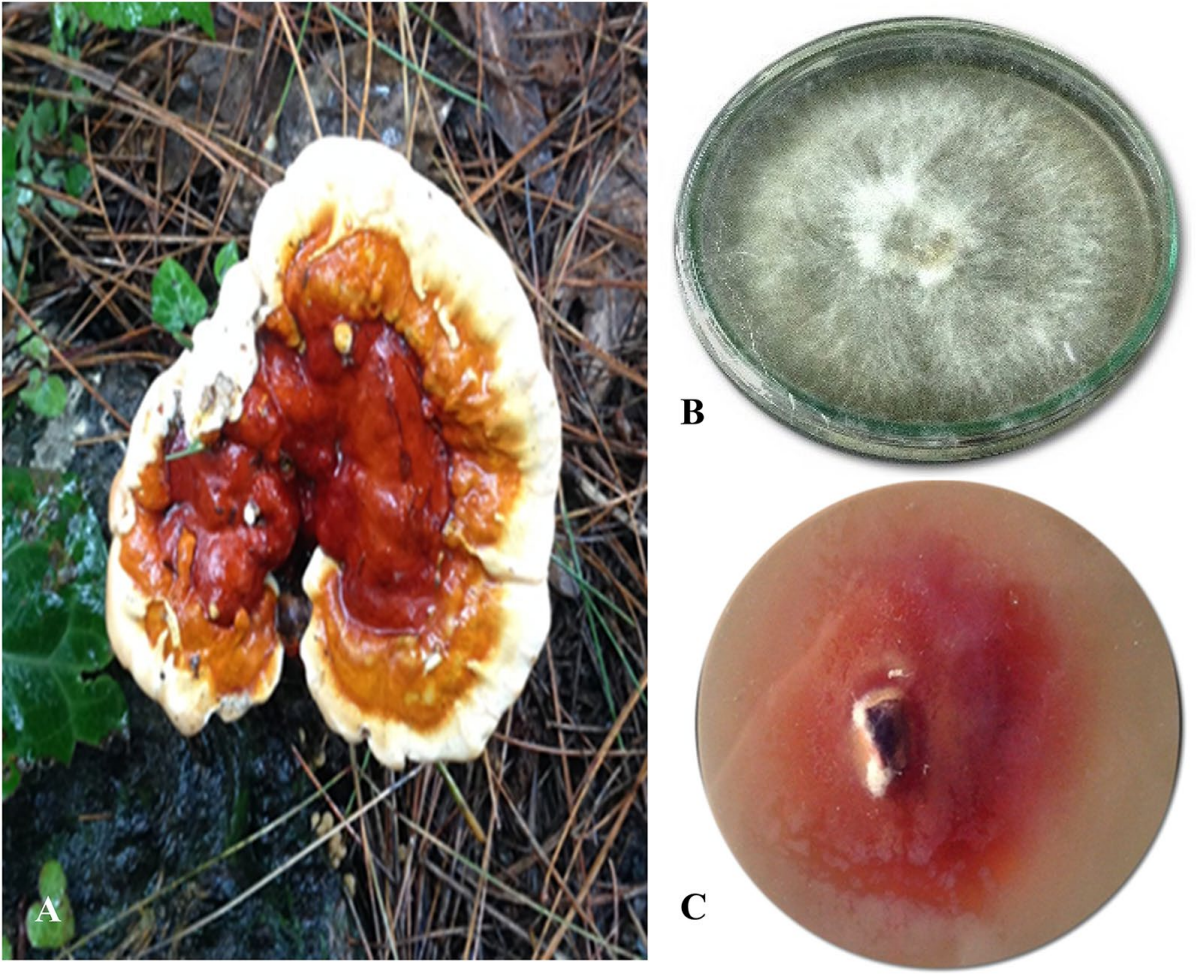
(**A**) *Ganoderma leucocontextum* Basidiome. (**B**) MEA Plate of Pure Mycelium. (**C**) Guaiacol Oxidase Halo (All photographs were taken by Aisha Umar).

*Ganoderma leucocontextum* was collected in monsoon season (June to August, 2018) from Pakistan, dried in a dehydrator, and kept in sealed polythene bags (Fig. 1A).

### Study site description

The specimens of *G. leucocontextum* (ANP1, AY2B) were collected from Ayubia National Park, Khyber Pakhtunkhwa, District Abbottabad and Naran Valley, District Mansehra, Pakistan, respectively. This park is located in the western Himalayas, north of Murree and South of Nathiagali (33°51′54.83″N 73°8′19.57″E). This area is covered by temperate broadleaf, mixed and temperate coniferous forest. The average temperature and rainfall is 3–11 °C and 1244 cm, respectively^23^.

### DNA extraction, sequence alignment and molecular phylogeny

A CTAB procedure was used to extract the DNA of the specimens^24^. The nuclear ribosomal regions were ITS1 and ITS4 used to study the target species^25^. Amplified PCR products were purified and sequenced by TSINGKE Co. Ltd. (China).

**Figure 2:**
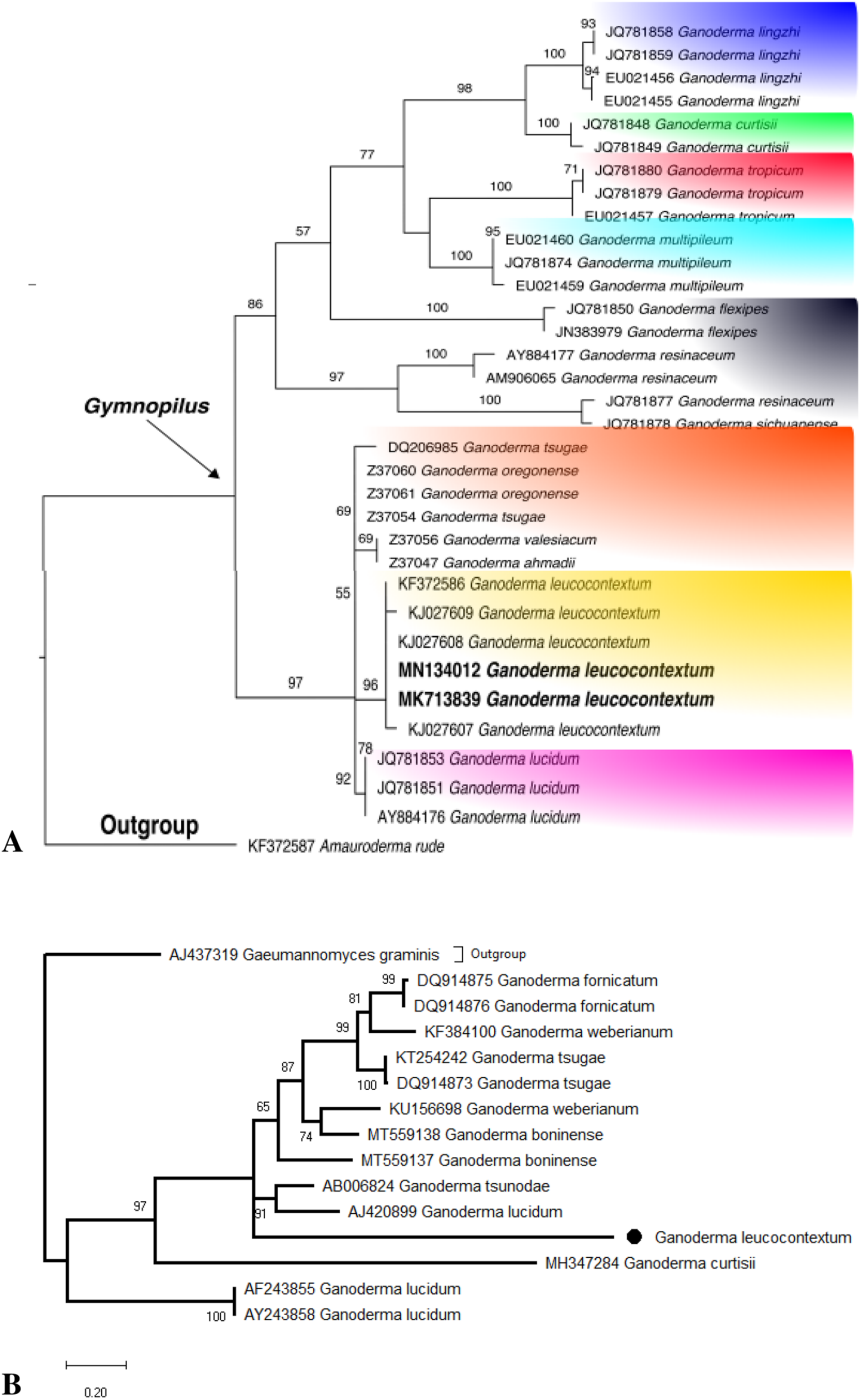
(**A**) Phylogenetic tree of *Ganoderma leucocontextum* and related species based on ITS sequences generated by maximum likelihood method in MEGA 10. *Amauroderma rude* was chosen as outgroup. Bootstrap values (> 50%) are shown at the branches (Constructed by Aisha Umar). (**B**) Phylogenetic tree of *Ganoderma leucocontextum* laccase and related species sequences generated by maximum likelihood method in MEGA 10. *Gaeumannomyces graminis* was chosen as outgroup. Bootstrap values (> 50%) are shown at the branches. Laccase of *G. leucocontextum* indicated by black dot (Constructed by Aisha Umar).

The consensus sequence was generated in BioEdit version 7.2.5^26^. The homology searches were performed at the NCBI using BLASTn. The sequences of this study were deposited in GenBank. The alignment was manually edited at 593 positions. The *Amauroderma rude* (Berk.) Torrend was used as an outgroup to support the tree. The MEGA 10.0 was used for phylogenetic analysis with 1000 replicates of bootstrap^27^ (Fig. 2A). The sequences under MK713839 and MN134012 accession numbers were deposited in the GenBank.

*Ganoderma leucocontextum* laccase genes were identified by degenerate primers accordingly D’Souza et al.^28^. The laccase genes of exoenzyme were confirmed by a guaiacol test. The primers Cu1F (5′-CAT(C) TGG CAT(C) GGN TTT(C)TTT(C) CA-3′) and Cu2R (5′-G G(A)CT GTG GTA CCA GAA NGT NCC-3′) exhibited the better results during amplification. The template of genomic DNA was isolated from fungal material for single PCR. The amplifications steps were comprised DNA extracts (3 µL) added to a reaction mixture (50 µL) containing of 10X Taq buffer (5 µL) with MgCl_2_, 10 mM dNTPs (4 µL), Taq DNA polymerase (0.2 µL) and 60 µM of both primers (1 µL). The nucleotide sequences of our specimens were deposited in NCBI under the mentioned accession numbers (GenBank MK713839; MN134012). The coding regions and protein sequences after alignment were carried out with identified laccase of different *Ganoderma* species. The deduced coding regions of sequence released and the additional laccase sequences were retrieved from GenBank, which manually aligned by BioEdit program. The phylogenetic tree was constructed by MEGA10 software using the Maximum Likelihood (ML) method with 1000 replications. The *Gaeumannomyces graminis* was used as outgroup to root the laccase tree (Fig. 2B).

The phylogenetic identification was confirmed by the author Aisha Umar. These specimens were submitted to “Lahore Herbarium (LAH)” of University of the Punjab.

### Qualitative analysis

Malt Extract Agar media was prepared in g/L by adding Malt Extract 7, Agar 10, MgSO_4_·7H_2_O 0.5, K_2_HPO_4_ 0.5, KH_2_PO_4_ 0.5, ZnSO_4_ 0.005, MnSO_4_ 0.05, Peptone 2.5 and Glucose 15^29^ at pH 5.0 (Fig. 1B). The streptomycin (200 mg/L) was added as an antibacterial agent. The above mentioned nutrient agar media was sterilized in an autoclave for 20 min at 121 °C. After autoclaving 0.02% guaiacol was added to MEA media to screen the best laccase producing specimens. All the plates were incubated at 30 °C for 5 days. The formation of reddish brown oxidation zone on agar plate media was used to screen the laccase from *G. leucocontextum.*

### Quantitative analysis

Kirk’s medium was designed for quantitative analysis of laccase activity with a little modification in the shake flasks. The macronutrients and tracer elements (g/L) of Kirk’s medium were taken in shake flasks for the growth of mycelium. The macronutrients with little modifications were mixed in g/L (glucose 10 g, yeast extract 5 g, starch 1 g, while tracers MgSO_4_⋅7H_2_O 0.5 g, NaCl 0.5 g, FeSO_4_⋅7H_2_O 0.5 g, KH_2_PO_4_ 0.046 g, K_2_HPO_4_ 0.1 g, CaCl_2_⋅2H_2_O 0.5 g, ZnSO_4_ 0.02 g, CuSO_4_⋅5H_2_O 0.5 g, H_4_PO_4_ 1.0 g, Na_4_HPO_4_ 0.05 g, MnSO_4_ 0.001 g, ZnSO_4_ 0.001 g^30^. The pH of liquid medium was adjusted to 5.0. The complete medium of 1 L shake flask was autoclaved and cool down before inoculum with mycelium discs.

Three mycelia plugs (5 mm diameter inoculum) of pure culture were added in the above mentioned autoclaved liquid medium (100 mL) of each flask and incubated at 27 ± 2 °C in static condition for 3 days. After 3rd day, culture medium was moved dynamically via shaker to optimize the growth and nutritional conditions. Add the ingredients one by one in continued culture till the optimization of each parameter achieved. The liquid samples in shake flask or submerged conditions were used for the analysis of laccase activity^31^.

The enzyme activity was determined by 100 mM guaiacol substrate dissolved in 100 mM sodium acetate buffer (pH 5.0). This reaction mixture contained 1.5 mL acetate buffer, 1.5 mL guaiacol and 1.0 mL of crude enzyme source. The laccase activity was measured at 27 ± 2 °C or room temperature after 15–30 min^32^. The changed in the absorbance of reaction mixture comprised guaiacol was monitored for 3 min at 470 nm by UV Spectrophotometer^33^. This activity was determined in triplicate by monitoring the absorbance for 3–5 min and expressed in U/L^34^.

$$\frac{{\text{U}}}{{\text{L}}} = \Delta {\text{Abs}}470*\frac{{{\text{Vt}}}}{{{\EUR}*{\text{l}}*{\text{Vs}}}}$$

where, € = 6740 M^−1^ cm^−1^ extinction coefficient of guaiacol, Vt = Total vol. of reaction mixture (mL), Vs = Vol. of the sample (mL), l = Length of cuvette (1 cm).

### Optimization of cultural and nutritional growth conditions for laccase production

Each culture flasks (100 mL) with few mycelia discs were incubated for 7 days at different temperature (20 °C, 40 °C, 60 °C) and pH (3.0, 5.0, 6.0). Each fermentation broth (100 mL) with a few mycelia discs was incubated for 7, 10 and 15 days to maximize the laccase production at different rpm (50, 100, 150) to maximize the laccase production.

Liquid medium of flask was modified by changing the concentration and nature of nutritional sources. Three actively grown discs from mycelium of *G. leucocontextum* were taken via cork borer and inoculated in 250 mL Erlenmeyer flasks contained liquid broth (pH 5.0). These cultural flasks were incubated on a rotary shaker (40 °C) at 100 rpm. After 10 days of incubation, the laccase activity was measured^35^. The liquid culture was decanted on Whatman No.1 paper, and the filtrate collected to optimize the following factors:

Different carbon sources (20 g and 25 g) like maltose, glucose and sucrose were evaluated for laccase production. The suitable inorganic and organic nitrogen sources were selected to check the maximum laccase production. The organic nitrogen sources included peptone, beef extract and yeast extract (5 g, 10 g), and inorganic ammonium sulphate, sodium nitrate and potassium nitrate were amended in the concentrations of 5 g/L and 10 g/L. The flasks were incubated for 10 days at 40 °C.

### Purification of laccase isozymes

A complete set up of culture broth (1000 mL) was designed separately under best optimized nutritional and growth condition. The broth was filtered through Whatman filter no. 1 and filtrate centrifuged at 13,000×*g* for 15 min at 10 °C. The supernatant was collected for partial purification of laccase. The finely grounded powder of (NH_4_)_2_SO_4_ was mixed thoroughly in cold supernatant till the saturation level was achieved (60% and 80%) for protein precipitation^36^. This saturated enzyme assay was incubated overnight at 4 °C and these precipitates were collected by centrifuging at 12,000×*g* for 35 min. After that the protein pellets were dissolved in 20 mM citrate–phosphate buffer (pH 5.0). The same buffer was used in dialysis at 4 °C for 1 day^37^.

### Laccase molecular weight

The yield of expressed protein was evaluated by SDS-PAGE, using a Criterion XT gel system (Bio-Rad, CA, USA). Estimated protein molecular weight (MW) of laccase was made against the standard protein markers (14.3–97.0 KDa). In order to assign ∼ 65 kDa laccase, a native PAGE was performed and stained with guaiacol. The separated protein was visualized by incubating the gel in 50 mM sodium acetate buffer (pH 5.0) containing 100 mM guaiacol.

### Biochemical characterization of laccase

The pH (2.0‒8.0) was maintained to examine the laccase activity and stability at 40 °C in 50 mM citrate phosphate buffer. The relative enzyme activity was taken after every 15 min. Temperature effected the laccase activity, which measured by incubating the protein at optimal pH. The temperature (10 °C to 80 °C) adjusted to determine the thermo-stability and readings were taken after every 10 min by increasing 10 °C temperature on each round. The effect of metal ions on laccase was determined to check the stability and relative activity. The metal ions (Cu^2+^, Ca^2+^, Zn^2+^) with sulfate donor in concentration of 1, 3, 6, and 9 mM was used in this study. The aliquot of enzyme, 50 mM citrate–phosphate buffer (pH 3.0), particular metal ion concentration was mixed throughly, and then incubated for 30 min at 40 °C.

A few inhibitors were examined by incubating the purified laccase for 30 min at room temperature. A control was run parallel without inhibitors to estimate the % inhibition on laccase activity and its performance. The concentration of SDS and EDTA was 1, 3, 6 and 9 mM, whereas 0.01, 0.05 and 0.1 mM of NaN_3_ investigated in this study.

### Kinetic studies

The kinetic parameters *K*_*m*_ and *V*_*max*_ of purified laccase isozyme was determined by using substrate at different concentration of 1 mM, 2 mM, 3 mM, 5 mM and 10 mM guaiacol in 100 mM citrate–phosphate buffer (pH 3.0).

### Statistical analysis

The data collected from various parameters during presented study was subjected to statistical analysis in computer software, Co-Stat version 3.01. Assays were carried out in triplicate and the values were presented as mean ± standard deviation.

### Consent to participate

I and my co-author participated equally.

### Consent for publication

I and my co-author allow the journal to publish my work.

## Results

### *Ganoderma* species identification

*Ganoderma leucocontextum* was identified by molecular method. The ITS-5.8S rDNA sequence of 570 bp was amplified from the genomic DNA. After NCBI blasting, the sequences with great identity were selected and used to generate the phylogenetic tree. It can be seen from the topology that specimens showed the maximum identity with *G. leucocontextum* (Fig. 2). The closely related species in the phylogenetic tree were *G. lucidum* (Curtis) P. Karst.*, G. oregonense* Murrill*, G. tsugae* Murrill.*, G. ahmadii* Steyaert. and *G. valesiacum* Boud. The bootstrap 96 (Fig. 2), where specimens (ANP1, AY2B) were nested in a well-supported clade of *G. lucidum* complex by forming a discrete lineage.

The phylogenetic identification was confirmed by the author Aisha Umar. These specimens were submitted to “Lahore Herbarium (LAH)” of University of the Punjab under the proper voucher/LAH number (LAH36345, GenBank MK713839; LAH36346, GenBank MN134012).

### Qualitative analysis

The laccase producing *G. leucocontextum* was preliminarily screened to produce the reddish brown halos on MEA plates (Fig. 1B,C) contained guaiacol indicator. This exhibited a biggest reddish brown colored zone around the colony after 7 days of incubation at 27 °C (Fig. 1B,C).

### Optimization of culture growth conditions

**Figure 3:**
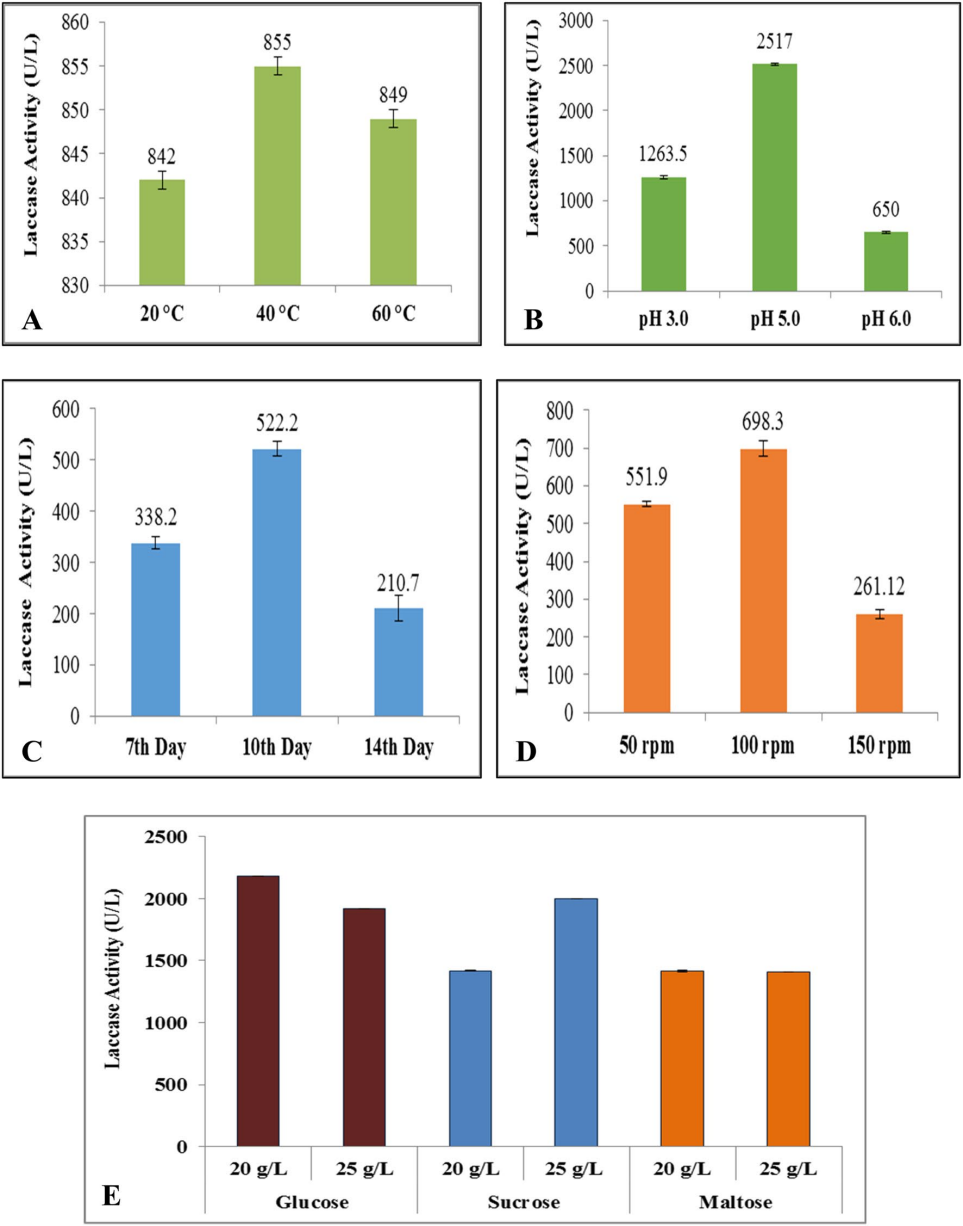

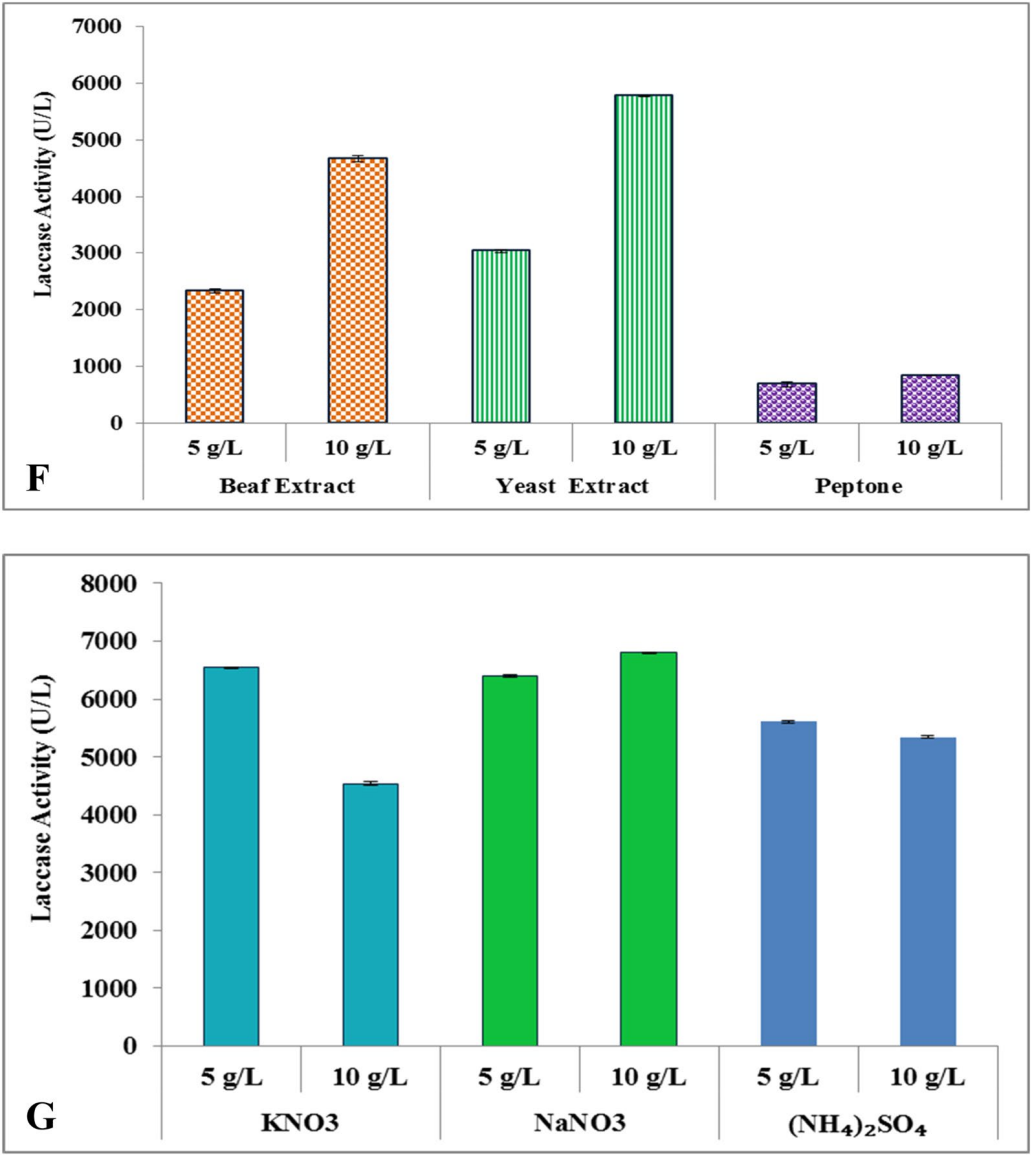
Optimization of cultural conditions in submerge fermentation broth of *G. leucococntextum.* (**A**) Optimization of T at 20 °C, 40 °C and 60 °C. (**B**) Optimization of pH at 3.0, 5.0 and 6.0. (**C**) Optimization of incubation periods on 7th, 10th and 14th days. (**D**) Optimization of rpm at 50, 100 and 150 rpm. (**E**) Optimization of organic 20 g/L and 25 g/L carbon sources. (**F**) Optimization of organic 5 g/L and 10 g/L nitrogen sources. (**G**) Optimization of organic 5 g/L and 10 g/L nitrogen sources. (**G**) Optimization of inorganic 5 g/L and 10 g/L nitrogen sources.

Individual culture flasks (100 mL) with few mycelia discs of *G. leucocontextum* were incubated for 7 days at different temperature (30 °C, 35 °C, 40 °C). The best optimized 40 °C exhibited maximum laccase production (855 U/L). At 20 °C, laccase production reduced as the temperature was increased from 40 to 60 °C (Fig. 3A).

The fermentation broth was inoculated with mycelia discs and incubated for 7 days at different pH (3.0, 5.0, 6.0). The suitable pH was 5.0 for guaiacol assay. The maximum production of laccase was 2517 U/L, whereas very low and higher acidity reduced the secretion level of laccase. So, the best suitable pH was 5.0, which enhanced the synthesis of laccase (Fig. 3B).

Optimum time of incubation required for the production of maximum laccase in this study. The incubations days were 14 in total. The harvested cultures were evaluated after 3 days interval. After 7 days, the secretion was enhanced (522.2 U/L) but decreased as the medium exhausted due to shortage of nutrients for mycelium after 10 days (Fig. 3C).

The 50, 100 and 150 rpm of orbital shaker was set to optimize the maximum laccase secretion and mycelium growth. The maximum production rate of laccase from *G. leucocontextum* was achieved at 50 and 100 rpm. The laccase activity 5551.9 U/L at 50 rpm and 698.3 U/L at 100 rpm was achieved in this work (Fig. 3D).

Organic carbon sources were selected to enhance the production of laccase including glucose, sucrose and maltose (20 g/L and 25 g/L) in fermentation broth contained mycelium of *G. leucocontextum*. The 20 and 25 g/L glucose has been exhibited the greater production of laccase than sucrose. The both concentrations of maltose showed less than 1500 U/L of laccse. So, glucose and sucrose were better to enhance the yield than maltose (Fig. 3E).

The extracellular enzyme from *G. leucocontextum* was studied in the presence of different organic and inorganic nitrogen sources. The organic nitrogen sources stimulated the more laccase production. Peptone, yeast and beef extracts were also evaluated (Fig. 3F). The optimal enzyme production was attained by using peptone nitrogen source. The 10 g/L beef and yeast extract was the best defined organic nitrogen source for laccase production *i.e.,* 4671 U/L and 5776 U/L, respectively from *G. leucocontextum* (Fig. 3F).

The effect of inorganic nitrogen sources (KNO_3_, NaNO_3_ and (NH_4_)_2_SO_4_) were determined by using 5 g/L and 10 g/L concentrations (Fig. 3G). The influence of inorganic nitrogen on laccase activity was dependent on the nature and concentrations. Opposite to this; KNO_3_ maximized the enzyme production (6540 U/L) at low concentration (5 g/L) rather than higher. It was apparent that 10 g/L KNO_3_ inhibited the enzyme formation. On other side, NaNO_3_ and (NH_4_)_2_SO_4_ caused the stimulation in laccase production. So, 5 and 10 g/L NaNO_3_ and (NH_4_)_2_SO_4_ was potent to enhance the laccase activity.

### Purification and identification of laccase isozyme

A complete set up of culture broth (1000 mL) was designed separately under the best optimized conditions. Filtrate was centrifuged at 13,000×*g* for 15 min at 10 °C and finely grounded powder of (NH_4_)_2_SO was mixed thoroughly in supernatant (60% and 80%) of filtrate. The best suitable concentration was 80% ammonium sulphate yielded 65% laccase. The laccase was purified 4.5-folds from its initial culture broth with a final yield (82%). The total activity of the purified enzyme was 15,228.5 ± 22.0 U/L by guaiacol (100 mM) assay.

The protein (Glacc 110) molecular weight of ∼ 65.0 kDa was estimated by SDS-PAGE (Fig. S1A) and Native PAGE (Fig. S1B). A single brown band of ∼ 65.0 kDa in a lane was stained by guaiacol, which indicated the presence of an active laccase of *G. leucocontextum* extract.

### Characterization of purified laccase

**Figure 4:**
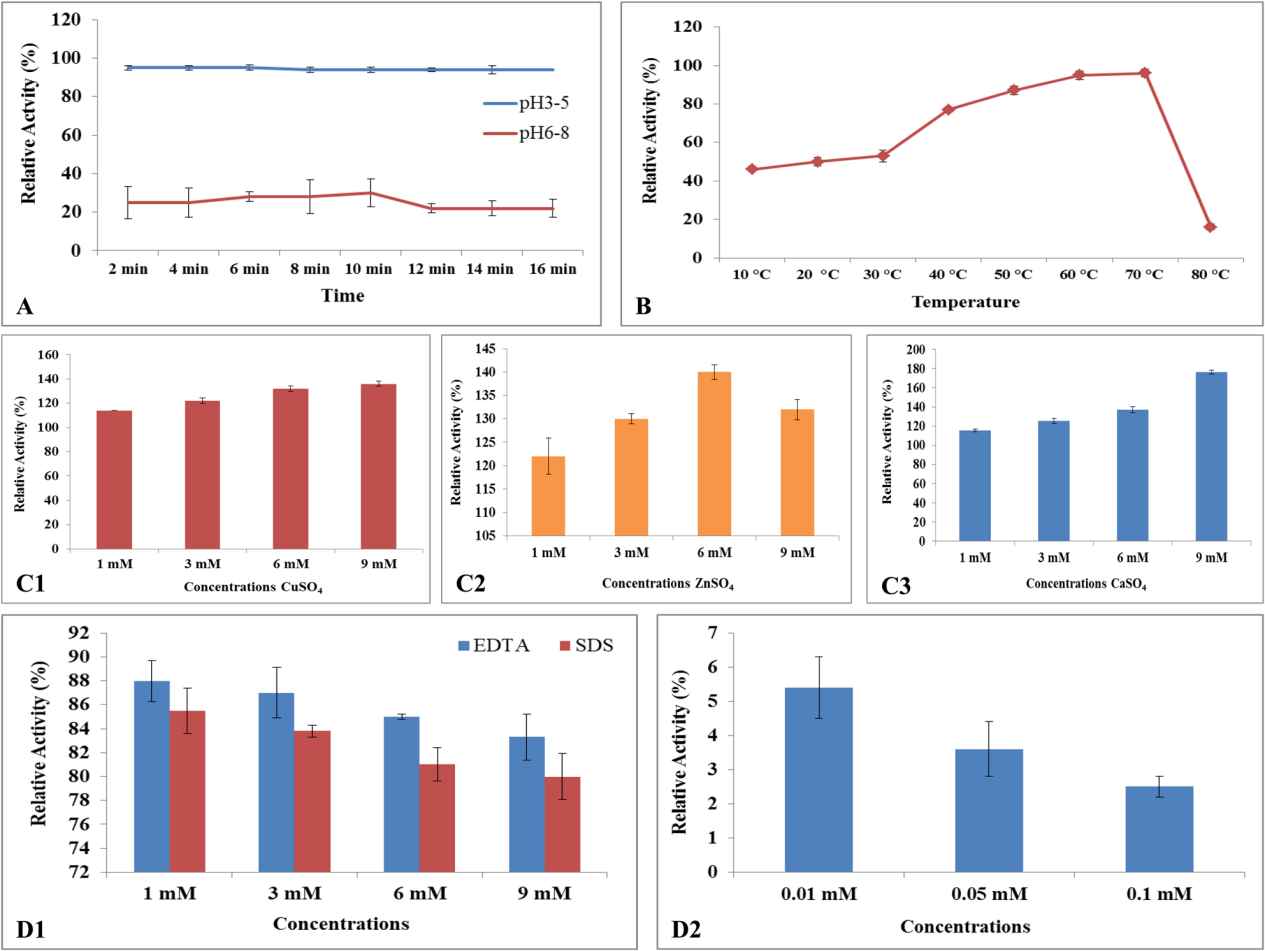
Characterization of purified Glacc110 extracted from *G. leucocontextum.* (**A**) Effects of pH ranges on purified Glacc. (**B**) Effects of T on purified Glacc110. (**C**) Effects of metallic ions on purified Glacc. (**C1**) Effects of CuSO_4_ (1, 3, 6, 9 mM) on purified Glacc110. (**C2**) Effects of ZnSO_4_ (1, 3, 6, 9 mM) on purified Glacc110. (**C3**) Effects of CaSO_4_ (1, 3, 6, 9 mM) on purified Glacc110. (**D**) Effects of inhibitors on purified Glacc110. (**D1**) Effects of EDTA and SDS on purified Glacc110. (**D2**) Effects of NaN_3_ on purified Glacc110.

Various buffers were tested to evaluate the laccase stability at different pH. The optimum pH to achieve the maximum activity of purified laccase was 3.0, while this enzyme also stable at pH 4.0. The relative activity was > 90% evaluated for 16 min. Readings were taken after 2 min time interval, whereas the activity dropped as the pH increased (Fig. 4A).

The temperature-dependent activity and thermal stability of purified laccase from *G. leucocontextum* showed optimum activity in sodium acetate buffer (100 mM, pH 3) at 70 °C. The higher temperature reduced the enzyme activity. The thermal stability of the purified laccase from *G. leucocontextum* was maximal at 40 °C to 60 °C, while decreased abruptly beyond 70 °C. The relative activity was 77% and 96% at 40 °C and 70 °C, respectively (Fig. 4B).

The effect of various metallic ions were evaluated on purified Glacc110 activity by adding Cu^2+^ (copper sulfate), Ca^2+^ (Calcium sulphate) and Zn^2+^ (zinc sulphate) to the reaction mixture of *G. leucocontextum* with control (100%) set. Various concentrations (mM) of metals were applied to characterize their effects. The CuSO_4_ (1, 3, 6, 9 mM) (Fig. 4C1), ZnSO_4_ (1, 3, 6, 9 mM) (Fig. 4C2), and CaSO_4_ (1, 3, 6, 9 mM) (Fig. 4C3) were applied, while the highest laccase production observed at 9 mM CuSO_4_ and CaSO_4_ (Fig. 4C1,C3), whereas 6 mM ZnSO_4_ was also effective (Fig. 4C2). The laccase production was significantly increased, when the culture medium was amended with 9.0 mM CuSO_4_ and CaSO_4_, but less increased at 9.0 mM ZnSO_4_ (Fig. 4C1–C3).

The effects of various inhibitors on purified Glacc110 activity was investigated by using SDS, EDTA and NaN_3_. The laccase activity was measured by pre incubating the purified Glacc110 in the presence of each inhibitor at 70 °C for 15 min. The 1 mM and 3 mM was effective to retain the maximum relative activity than 6 mM and 9 mM of EDTA. The relative activity was 88% and 87% at 1 mM and 3 mM of EDTA, respectively.

SDS was a bad inhibitor for the laccase activity than EDTA at all concentrations. The NaN_3_ was also a bad inhibitor for laccase activity at all the concentrations used in this work rather EDTA and SDS (Fig. 4D1,D2) and negligible relative activity exhibited by Glacc110.

### Kinetic studies

**Figure 5:**
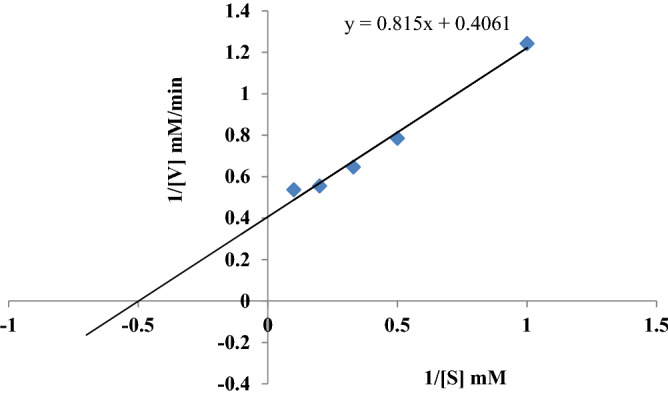
The Lineweaver–Burk plot of purified Glacc110 of *G. leucocontextum.*

The time course of the oxidation of guaiacol in the presence of purified laccase is shown in this study (Fig. 5). The kinetic constants of purified *G. leucocontextum* laccase were determined in this study. The *K*_*m*_ and *V*_*max*_ of *G. leucocontextum* laccase was 1.658 (mM) and 2.452 **(**mM/ min), respectively.

## Discussion

The factors leading to taxonomic confusions are morphological features, inter hybridization, geography, abiotic factors, and genetic heterogeneity^38,39^. Phylogenetic reconstructions of DNA sequences increased the reliability of evolutionary framework. This powerful data answered the ambiguous questions related to species identification^40^. Our new *Ganoderma *record (ANP1, AY2B) was grouped well within the sequences of Chinese *G. leucocontextum*^4,22^.

Guaiacol is a reliable and easily detectable screening step of laccase activity. The halo formed due to oxidation of indicator via lignolytic enzymes^41^. The production of intense brown, brown, and reddish brown color under and around the fungal colony was considered the positive result of guaiacol oxidation^42^. The obtained results had good agreement with Kiiskinen et al^41^. Temperature is a significant environmental factor to regulate the secretion of laccase isozymes^43^. This is fungal dependent parameters that stimulate the enzyme production^44^. Industrial applications demand not only the optimal temperature, but need to measure the robustness of laccase at higher temperature. Laccase in fungi act as phenol oxidases prefer the temperature range of 30–55 °C to get catalytic activity^45^, which varies under the optimal temperature (30 °C and 55 °C)^46^. The fungal activity of enzyme was condensed or minimized, when cultivated above 30 °C^47^. The maximum enzyme production under high temperature indicated an adaptive step of white rotting basidiomycetes to grow and survive in hot nature of environment.

The pH variation is an important factor for the improvement of laccase quantity^48^. Fungal laccase exhibited higher stability in acidic pH (pH 4–6/3.6–5.2) to promote the catalytic efficiency^45,49^. The mobility of polypeptide chain increased electrostatic interactions at pH 3.0. Low pH causes the unfolding of protein due to accumulation of hydrophobic surfaces. This was a logical hint to loss the laccase activity^50^. The maximum laccase quantity in a shorter period is advantageous in industrial applications, while fungal species require longer period for laccase production^51^. In the same way, researchers prefer 14 to 20 days for maximum laccase secretion from wood rotting fungi^52^.

The fungal mycelium biomass covered the liquid medium on 3rd day of incubation to secrete the maximum laccase. But this view about *Gaonderma lucidum* was contradicted to our results^48^. In this study, mycelium biomass fully covered the broth on day 6th and 7th. The reported laccase activity was 27 U L^−1^ of *Ganoderma* sp.^53^, 80 U L^−1^ of *G. australe*^54^, and 120 U L^−1^ from *Ganoderma* sp. En3^55^. Rodrigues et al.^10^ used 3 fungal discs (5 mm) of *G. lucidum* on PDA medium and incubated at 27 °C for 8 days at 150 rpm for maximum production of laccase. In this work, *G. leucocontextum* formed filamentous mats due to restriction of oxygen between fungal mycelium and medium. The similar views were represented by Madhavi and Lele^55^. The earlier studies showed the highest laccase production in agitated cultures^56^.

The carbon sources are significant inducers in production of laccase^57^. These sources symbolized the first sign of mycelial growth within 24 h, while completely colonized within 6 days^58^. The excessive glucose concentration was inhibitory to extracellular laccase production from several *Ganoderma* species^59^. Li et al.^60^ given two opinions to the scientist: (1) glucose is a strong inhibitor of laccase expression from *Ganoderma* isolates or (2) glucose is an important nutrient to convince the *G. lucidum* to secrete the laccase^60^. The activity of laccase is dependent on concentration, nature of carbon sources and mushroom species^61^. Presence of sugar reduced the enzyme yield by repress the catabolites^62^. This repression is associated with laccase expression^63^. Several authors reported low carbon–nitrogen ratio^64^, while a few prefer the high carbon–nitrogen ratio^65^. In basidiomycete, high concentrations of glucose inhibit the laccase transcription^66^. The excess of any nutrient cause the blockage in induction, and permit the constitutive enzyme production. Sugar supported the sufficient mycelium biomass formation but not the guarantor of maximum enzyme yields^67,68^. The laccase activity is dependent on concentration and nature of nitrogen sources in wood rotting fungi^67–69^. The organic nitrogen sources are more efficient than inorganic^10^. The nitrogen was not affected the enzyme activity and yield of some fungal species^9^. The concentrations of nitrogen suppressed as well as stimulate the ligninolytic enzyme activity in several species e.g., *Trametes trogii*^69^.

The kDa of laccase ranges are 24–85 kDa^70^, 50–80 kDa^71^, 55–90 kDa^72^, 50–100 kDa^73^, 40–66 kDa^74^, 38.3 kDa from *Ganoderma* sp. KU-ALK4^57^, 38 and 60 kDa from *T. trogii*^69^, 45 and 90 kDa from *C. versicolor*^75^, 61.7 kDa from *Mycena purpureofusca*^76^, 66 kDa from *Lentinus squarrosulus*^77^ and *Thelephora terrestris*^78^, 65 kDa from *Trametes* sp. LS-10C^44^, 67 kDa from *P. ostreatus*^79^, 59 kDa from *Pleurotus sajor-caju*^80^, 70 kDa from *Phellinus linteus*^81^, 75 and 150 kDa from *T. villosa*^75^. The LacI and LacII isozymes of 66 kDa were determined by SDS-PAGE from *Coriolopsis rigida*^82^.

Zou et al.^83^ explained that many fungal laccases are functional under acidic or neutral pH and lost their functionality under alkaline conditions. They found the laccase activity at pH 5.0 after 24 h incubation. Laccase of *G. lucidum*-CDBT1 was most stable at pH 5.0 and 30 °C^84^. The preferable stable acidic pH region was 5.0 in *Hericium erinaceum*^85^ and *Lentinula edodes*^86^. Purified laccase of *Ganoderma* sp. was active under acidic pH (3.0–5.5), stable at pH 3.0 to 5.0 and maximum activity was found at pH 4.5^33^. Purified laccase retained 95% residual activity at pH 5 and 50% at pH 6.0^33^. Rate of laccase inactivation increased with increase in temperature from 10 to 60 °C, while stable at 10 to 30 °C^87^. Thermostabilty depend upon time and substrate *e.g.,* laccase at 60 °C was stable for 24 h extracted from *C. gallica*^88^, 5–9 h of *Peniophora* sp., and 10 min in *T. gallica*^89^. The residual laccase activity of *G. lucidum* was maintained and examined every 10 min for 80 min at pH 3.0, when incubated under 60 °C^90^. The laccase was very stable at pH 6.0 and 7.0, whereas moderately stable at pH 5.0 and 8.0 (25 °C) of *T. versicolor*^91^. Temperature greater than 60 °C dropped rapidly the activity of fungal laccase^92^. The laccase activity increased smoothly from 30 to 60 °C (sharply increased at 50 °C and 60 °C), while decreased over 80 °C in *Echinodontium taxodii*^93^. In this study, favorable highest temperature range was 50 °C to 60 °C. Similarly more than 70% laccase activity of Glac15 was maintained at 50 °C by guaiacol substrate^94^, whereas 25–35 °C significantly reduced this activity^79^.

Fonseca et al.^43^ produced the highest laccase activity at 0.5 mM Cu^2+^ in culture medium of *G. applanatum* and *Peniophora* sp. The enzyme production and fungal growth was inhibited under the higher concentration of Cu^2+^ (3 mM)^95^. The optimal concentrations of Cu^2+^ were 0.1 and 0.5 mM enhanced the laccase from *P. ostreatus* and *Streptomyces lavendulae*^96^, respectively. In fungal organisms, copper ions are essential to form the intact and active structures of laccase, whereas many reports showed that copper ions badly affect the laccase production ability^95^. Copper induction also influences the genetic transcription level of laccase^96,97^.

Ions (K^+^, Ca^2+^, Fe^2+^, Ba^2+^, Fe^3+^ Zn^2+^or Al^2+^) closely bind the T1 site of laccase. These ions perform a function like competitive inhibitor for e^–^ donors by hindering the access of substrates to the T1 site or stop the e^–^ movement to T1 active site. This action leads to inhibition of laccase activity^98^. The NaN_3_ is a typical laccase inhibitor, which bind to the type II and III Cu sites. The NaN_3_ binding affects the internal electron transfer, thereby inhibit the laccase activity^99^. Contrary, SDS enhanced the enzyme activity, which also changes the structure of enzyme^100^. The purified laccase was sturdily inhibited by SDS at 0.5 mM (13%) and 1 mm (6%) of *Trametes* sp. LS-10C^64^. Vantamuri and Kaliwal^101^ supplemented 20 mM EDTA in the purified enzyme of *Marasmius* species BBKAV79, while Das and associates^36^ determined the laccase activity from *Pleurotus florida*. This was interesting to know that 1 mM EDTA represented 116% inhibition rate of recombinant laccase from *T. versicolor*^20^.

The *V*_*max*_ is dependent on enzyme concentration^102^. Except slow oxidation, wood rotters laccase exhibited low affinity and catalytic constants (*K*_*m*_) with guaiacol. These are higher than other substrates found in this study. The *K*_*m*_(mM) value of purified laccase was 2.50 of *Pleurotus sajor-caju* and 0.107 of *G. lucidum* GaLc3^103^. The higher *K*_*m*_ (0.107 mM) value of laccase from *G. lucidum* was indicated the low enzyme affinity to the substrate^104^. The published value of *K*_*m*_ from wood rotters were 1.2 mM for *P. ostreatus*^105^, 0.550 for *P. pulmunarius* Lcc2 (mM)^106^, 2.81 mM for *P. florida*^36^, 2.095 mM for *P. rivulosus* Lac-3.5, 1.406 mM for *P. rivulosus* Lac-4.8^107^, 1.1250 mM for *Pycnoporus* sp*.* SYBC-L1 LacI, 0.7452 mM for *Pycnoporus sp.* SYBC-L1 LacII^108^, 0.917 mM for *Lentinula edodes*^86^*,* and 0.25 mM for *P. sanguineus*^109^.

## Conclusion

*Ganoderma leucocontextum* was identified by ITS markers. In this work, first time laccase was purified and characterized at the industrial level from *G. leucocontextum.* In conclusion, this study reported that laccase produced from *G. leucocontextum* cultures shown the isozymes by SDS and Native-PAGE with interesting properties like stability at higher temperature and acidic pH. There are still many wood rotting mushrooms/fungi, which not described till yet. The new researchers have to explore the maximum new diversity of genus *Ganoderma* with their biochemical characterizations and laccase production. This laccase is suitable for industrial and biotechnological applications. The few challenges for future researchers are the use of this laccase as a biocatalyst offer economically feasible domino processes for the preparation of bioactive compounds, immobilization of laccase, immobilization methods, evaluation methods for laccase activity, factors affecting the laccase expression, and heterologous expression.

## Supplementary Information

Supplementary Figure 1.

## Data Availability

The data set generated and analyzed during the current study are available at institute of plant sciences, University of the Punjab.

## Abbreviations

ITS: Internal transcribed spacer
U/L: Enzyme activity unit/L
EA: Enzyme activity
CTAB: Cetyl trimethylammonium bromide
BLAST: Basic local alignment search tool
NCBI: National Center for Biotechnology Information
MEGA: Molecular evolutionary genetics analysis
