# Supplementary material for: Optimization, purification and characterization of laccase from Ganoderma leucocontextum along with its phylogenetic relationship
Source: Sci Rep. 2022 Feb 14;12:2416. doi: 10.1038/s41598-022-06111-z (PMC8844424; doi:10.1038/s41598-022-06111-z)
Supplement: Supplementary file 1 — Supplementary Figure 1. [file 41598_2022_6111_MOESM1_ESM.pdf]

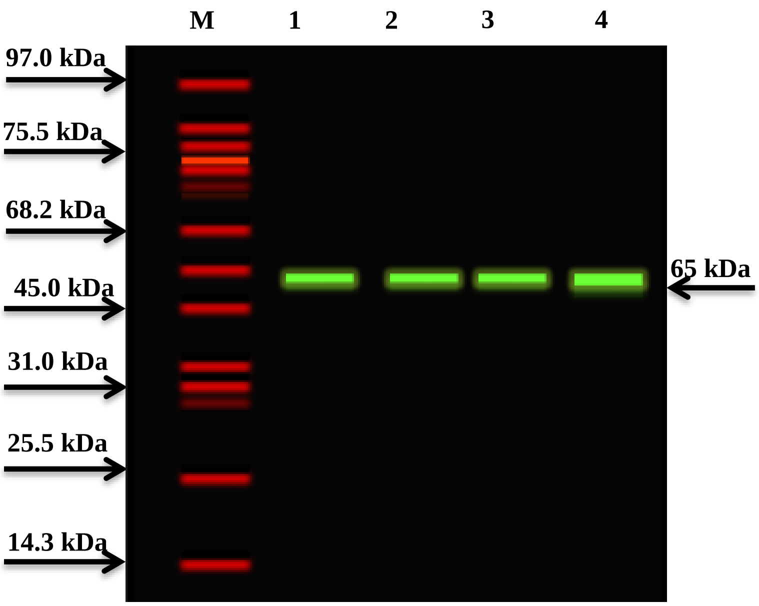

Fig. S1. A. Purified **Glacc110 (65.0 kDa)** of *G. leucocontextum* Identified by SDS-PAGE (Digital Image).

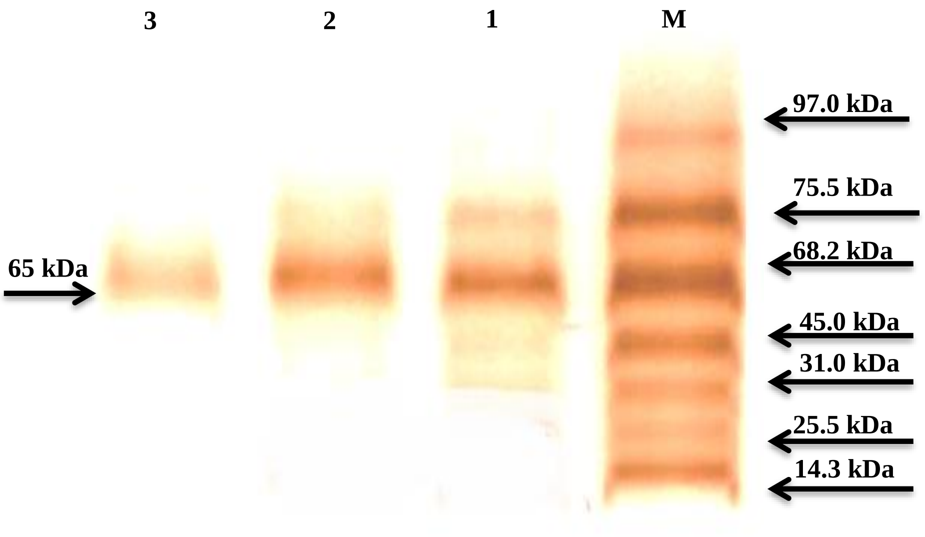

Fig. S1. B. Purified **Glacc110 (65.0 kDa)** of *G. leucocontextum* Identified by Native Page.
